# The Insulin-Like Growth Factor System in Obesity, Insulin Resistance and Type 2 Diabetes Mellitus

**DOI:** 10.3390/jcm3041561

**Published:** 2014-12-22

**Authors:** Moira S. Lewitt, Mairi S. Dent, Kerstin Hall

**Affiliations:** 1 School of Health Nursing & Midwifery, the University of the West of Scotland, Paisley PA1 2BE, UK; E-Mail: Mairi.Dent@uws.ac.uk; 2 Department of Molecular Medicine and Surgery, Karolinska Institutet, Stockholm SE171 76, Sweden; E-Mail: Kerstin.Hall@ki.se

**Keywords:** insulin-like growth factor, IGFBP, GH, obesity, type 2 diabetes

## Abstract

The insulin-like growth factor (IGF) system, acting in concert with other hormone axes, is important in normal metabolism. In obesity, the hyperinsulinaemia that accompanies peripheral insulin resistance leads to reduced growth hormone (GH) secretion, while total IGF-I levels are relatively unchanged due to increased hepatic GH sensitivity. IGF-binding protein (IGFBP)-1 levels are suppressed in relation to the increase in insulin levels in obesity and low levels predict the development of type 2 diabetes several years later. Visceral adiposity and hepatic steatosis, along with a chronic inflammation, contribute to the IGF system phenotype in individuals with metabolic syndrome and type 2 diabetes mellitus, including changes in the normal inverse relationship between IGFBP-1 and insulin, with IGFBP-1 concentrations that are inappropriately normal or elevated. The IGF system is implicated in the vascular and other complications of these disorders and is therefore a potential therapeutic target.

## 1. Introduction

Throughout evolution the insulin-like growth factors (IGFs), their IGF-binding proteins (IGFBPs) and receptors have had central roles in growth, metabolism and reproduction [1]. There is evidence that the IGF system has an important pathophysiological role across the spectrum of obesity, insulin resistance and type 2 diabetes mellitus, and therefore represents a potential therapeutic target. The purpose of this review is to present a short summary of that evidence.

Articles included were retrieved through PubMed using the MeSH terms “insulin-like growth factor” or “IGFBP” and identified by a manual search for English-language, full-text papers that related to obesity, insulin resistance and diabetes mellitus for the period 2009 to April 2014. In addition one of the author’s own database of IGF and IGFBP references prior to 2009 was searched using the terms obesity, insulin resistance and diabetes. Reference lists of papers identified further articles. Where possible earlier reviews are used. Although human studies were the primary focus, animal models that provide unique insights are also included.

An understanding of the role of the IGF system in obesity, insulin resistance and diabetes requires an in depth knowledge of its role in normal metabolism. This will be described first, after which the recent IGF literature in relation to obesity, insulin resistance and type 2 diabetes will be reviewed in the context of previous work.

## 2. Role of the IGF System in Normal Metabolism

### 2.1. IGFs and IGF Receptors

The IGF system is ubiquitous, with paracrine and endocrine metabolic roles. Structurally similar to proinsulin due to shared ancestry [2], the IGFs interact with insulin receptor (IR) A and B isoforms, the type 1 IGF receptor (IGF1R), and hybrid receptors (IRA-IGF1R and IRB-IGF1R) to mediate signals in variety of tissues to coordinate protein, carbohydrate and fat metabolism [3]. Hepatocytes and mature adipocytes have abundant IRB: insulin, having an affinity for IRB that is two orders of magnitude greater than IGF-I, plays the dominant metabolic role in these tissues. Preadipocytes have abundant IGF1R: IGF-I, having an affinity for IGF1R that is two orders of magnitude greater than insulin, mediates preadipocyte proliferation, differentiation and survival [4]. There is a shift from the expression of IGF1R to IRB during adipocyte differentiation. In muscle both insulin receptors and IGF1R are present, so that hybrid receptors contribute to metabolic signalling. It is recognised that IGF-II, which binds to IRA with similar affinity to insulin [5], may have particular metabolic roles. The type 2 IGF/mannose 6-phosphate receptor (IGF2R) sequesters IGF-II, thus influencing its availability for signalling. The extracellular domain of IGF2R exists as a soluble IGF-II-binding form in the circulation [6]. Preptin, derived from the E-peptide of pro-IGF-II, has been identified in mice as a pancreatic islet beta-cell hormone that is co-secreted with insulin in response to glucose and which increases glucose-mediated insulin secretion [7]. Whether preptin plays a significant role in human physiology is not yet known.

**Figure 1:**
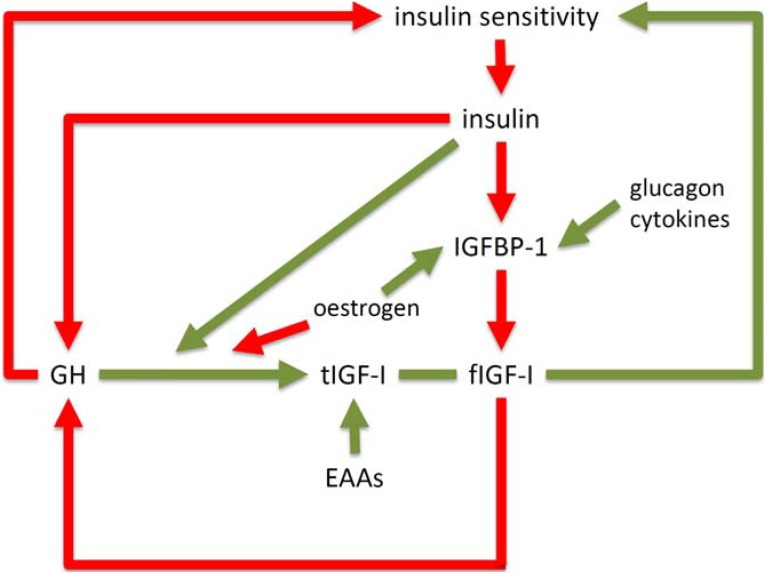
Interaction between insulin and the insulin-like growth factor system*.* Inhibitory effects are shown in red and stimulatory effects, in green. GH = growth hormone; tIGF-I = total insulin-like growth factor-I (unbound and in ternary and binary complexes with IGF-binding proteins (IGFBPs)); fIGF-I = free IGF-I; EAAs = essential amino acids.

IGF-I synthesis is stimulated by nutrition and growth hormone (GH) in liver and other tissues. There are gender differences in the hepatic sensitivity to GH: women require more GH for a similar effect [8]. IGFs that reach the pituitary inhibit GH synthesis in a feedback loop (Figure 1). GH has important metabolic roles that are independent of IGF-I effects, including stimulation of lipolysis and inhibitory effects on insulin signalling in fat and muscle [9]. Thus IGF-feedback inhibition of GH, by reducing these direct metabolic effects, will increase insulin sensitivity. In this way partially processed forms of proIGF-II secreted by large mesenchymal tumours and reaching the circulation, cause a syndrome of tumour hypoglycemia that is characterised by low circulating GH and total IGF-I concentrations [10]. Alongside and complementing insulin, IGFs directly regulate protein, carbohydrate and fat metabolism. IGF-I also enhances insulin sensitivity, independently of its effect on GH [3].

### 2.2. IGFBPs

In humans there are six IGFBP genes encoding a family of highly conserved proteins that have high affinity binding for the IGFs [11]. Two of these, IGFBP-3 and IGFBP-5, when bound to IGF-I or IGF-II, can bind to a third protein, the acid-labile subunit (ALS). ALS and IGF-I are growth hormone dependent and the high molecular mass ternary complex, since it is retained in the circulation, accounts for stable IGF-I concentrations in the face of pulsatile GH secretion. Unbound or “free” IGFs (fIGFs) and IGFs in binary complexes have short half-lives in the circulation, measured in minutes to hours [12]. Total IGF measurements in single blood samples therefore underestimate this dynamic IGF turnover and fail to reflect tissue IGF production, which contributes to activity at the cellular level. Determination of fIGF-I concentrations in interstitial fluid, for example, reveals concentrations that are higher than fIGF-I in plasma [13]. There are also a number of IGFBP proteases that are tissue-specific and reduce or abolish IGF-binding affinity, thus adding further complexity to the system.

It has long been realised that IGFBP-1 has an endocrine role in metabolism [14]. IGFBP-1 is secreted primarily from hepatocytes where synthesis is inhibited by insulin and stimulated by multiple regulators including glucagon, oestrogen, glucocorticoids, and pro-inflammatory cytokines [15]. Therefore circulating IGFBP-1 concentrations reflect the combination of the inhibitory effect of portal insulin, the degree of hepatic insulin sensitivity and the actions of various stimulators [16]. IGFBP-1 concentrations are higher in women than men [17]. Phosphorylated forms of IGFBP-1 acutely inhibit IGF effects on 3T3-L1 preadipocytes and adipocytes [18]. Specific proteolysis of IGFBP-1 generates IGF-binding fragments with reduced IGF-II-stimulated glucose uptake in muscle [19].

The IGFBP family has other metabolic roles through IGF-independent as well as IGF-dependent mechanisms. Studies in 3T3-L1 cells have demonstrated that IGFBP-3 inhibits adipogenic differentiation through direct interaction with PPAR-gamma [20] and IGFBP-2 expression in 3T3-L1 adipocytes is upregulated by insulin [21]. Studies in animal models show that IGFBP-3 knockout increases adiposity [22] and that IGFBP-3 also affects insulin secretion directly by IGF-independent as well as IGF-dependent mechanisms [23]. IGFBP-2 is also regulated by changes in nutritional status and it is speculated that IGFBP-1 and IGFBP-2, which both have Arg-Gly-Asp integrin recognition motifs, also modulate insulin sensitivity in an IGF independent manner [15,24]. It has been shown that IGFBP-2 is regulated by leptin and may mediate a portion of leptin’s antidiabetic effects [25]. Subcutaneous adipose tissue from lean women has been shown to secrete predominantly IGF-II and IGFBP-4 [26] while in omental preadipocytes secrete PAPP-A, which cleaves IGFBP-4 [27].

## 3. Obesity

Obesity is a complex metabolic disorder that is characterised by an increase in white adipose tissue mass [9]. Clinically it is defined as a BMI of ≥30 kg/m^2^, however this definition fails to take into account gender, differences in muscle mass, and the relative amounts of peripheral and central fat in an individual subject. Since central or abdominal obesity is an independent marker of cardiovascular morbidity and mortality [28], waist measurements, corrected for height, are recommended as a surrogate marker of central obesity that is better than BMI. Unfortunately many of the studies of the IGF system fail to include a measure of central adiposity and focus on BMI measurement.

### 3.1. Role of the IGF System in Obesity

Obesity is associated with reduced spontaneous and stimulated GH secretion that is reversible with weight loss [29,30]. GH deficient patients have central adiposity that is reduced by GH replacement, and it is therefore proposed that the suppressed GH level in obesity is in part responsible for a shift to relatively greater visceral adiposity [9]. The GH/IGF system inhibits activity of the enzyme 11β-hydroxysteroid dehydrogenase 1 which catalyses the conversion of cortisone to cortisol, and excess local cortisol production may partly contribute to central adiposity in both GH deficiency and obesity [31].

There are several hypotheses as to why there is suppression of GH secretion in obesity. It may be a consequence of suppressed IGFBP-1 due to increased portal insulin concentrations in response to peripheral insulin resistance (Figure 1). According to this hypothesis changes in IGFBP-1, by inversely regulating free IGF availability, alter the feedback inhibition of GH secretion. Consistent with this idea, IGFBP-1 levels are known to correlate inversely with insulin concentrations and measures of adiposity [16,17,32,33]. However, while it has been reported that fIGF-I levels are high in obesity [34], other studies show that fIGF-I is not increased [35] or may even decrease [36]. In an experimental setting, a two-week period of overeating was associated with increases in insulin, decreased IGFBP-1, increased fIGF-I and reduced GH [37]. However most of the decline in GH concentrations occurred before the increase in fIGF-I in the circulation. Although this might suggest the process is independent of IGFBP-1, it is still possible that lower concentrations of IGFBP-1 in tissues has an effect on fIGF-I action at the cellular level. In a study of obese individuals before and after weight loss, GH secretion normalised while fIGF-I levels increased [29]. Differences in assay techniques for fIGF-I may also contribute to the differences observed in some of these studies.

Recent work suggests that insulin may have a unique inhibitory effect on GH secretion. In mouse pituitary cells insulin inhibits GH, independently of IGFIR [38]. It appears likely that sensitivity to insulin in obesity is preserved in some tissues where it plays a central role in the GH-IGF axis response, by inhibiting pituitary GH, increasing hepatic GH responsiveness and suppressing hepatic IGFBP-1 secretion [30,39].

Obesity is associated with an increased IGF-I response to GH [39,40], and increased GH-binding protein levels [39], so that an increase in expression of GH receptor may explain lack of suppression of total IGF-I levels. Circulating total IGF-I concentrations in simple obesity are reported as low, normal or high [29]. Some studies report an inverse relationship between total IGF-I concentrations and measures of adiposity, such as waist circumference corrected for height [32,41]. Total IGF-I concentrations are also lower in thin individuals; and a non-linear U-shaped relationship between BMI and IGF-I has been reported in a population aged between 41 and 70, with highest IGF-I levels at BMI 24–26 kg/m^2^ [42]. A large study of adults in which IGF-I levels were adjusted for age also documented a U-shaped relationship: the relationship with BMI was positive in normal weight and negative in obese individuals. BMI accounted for a small effect, just 1%, of the variance in IGF-I in normal weight subjects, and this increased to 7% in obese individuals [43].

The relationship between IGF-II and obesity is even less certain. Increased tissue IGF-II expression in pigs results in a lean muscular phenotype [44]. In Silver Russell syndrome *Igf2/H19* hypomethylation is associated with insulin resistance and a phenotype of impaired fasting glucose in the presence of low BMI [45]. Low IGF-II concentrations have been shown to predict weight gain and obesity in adults with [46] and without [47] type 2 diabetes. In children low IGF-II levels have been documented in obesity, along with lower IGFBP-2 concentrations, particularly in those with insulin resistance and evidence of chronic inflammation (increased IL-6 and TNF-alpha) [48].

Childhood obesity is now a global health problem and there is a growing body of evidence of the role of the IGF system. In obese children there are strong inverse relationships between IGFBP-1 concentrations and insulin resistance, as well as the metabolic syndrome [49]. In a Hong Kong study higher IGF-I and IGFBP-3 concentrations were observed in obesity and in relation to cardiovascular risk markers in adolescence [50]. In contrast another study has shown decreased total IGFBP-3 in obese adolescents and increased circulating IGFBP-3 protease activity and IGFBP-3 fragments [51]. Since IGFBP-3 fragments have reduced affinity for IGFs and may have altered IGFBP-3 receptor binding, the impact of these changes on both IGF-dependent and IGF-independent metabolic effects should be considered in future work.

### 3.2. Metabolic Syndrome and Disease Risk

Insulin resistance and other features of the metabolic syndrome commonly accompany obesity. However the degree of peripheral and hepatic insulin resistance varies between individuals, as does the presence of chronic inflammation, as evidenced by markers such as proinflammatory cytokines and CRP. There are important genetic determinants that play a role in the accumulation of liver fat (hepatic steatosis) and development of mitochondrial dysfunction and adipose tissue inflammation in response to obesity. Obese twins that are metabolically “healthy”, for example, have similar insulin levels to their lean monozygotic twin pairs [52].

Genetic differences may also be responsible for variability in IGF system responses to obesity. Patients with Prader-Willi syndrome (PWS) have severe obesity and low GH, IGF-I and fIGF-I concentrations despite non-suppressed IGFBP-1 levels [53]. It is speculated that ghrelin, which is decreased in simple obesity but increased in PWS, might play a role in blocking the inhibitory effect of insulin on IGFBP-1 production [54]. Individuals with GH insensitivity syndrome due to a range of GH receptor mutations are also reported to be obese [55] and GH receptor knockout mice are insulin sensitive despite obesity and have an increase in subcutaneous fat mass and not hepatic fat [9].

Accumulation of hepatic fat (steatosis) is closely linked to hepatic insulin resistance and predicts the development of metabolic syndrome, type 2 diabetes and cardiovascular disease [56]. Hepatic steatosis is associated with reduced GH and IGF-I levels [57,58,59] and there is an inverse relationship between IGF-I levels and concentrations of inflammatory biomarkers [59,60,61] and fibrosis score [61] in these subjects. In abdominally obese men, treatment with GH improves body composition, including liver fat, mitochondrial function and markers of cardiovascular risk [62].

**Figure 2:**
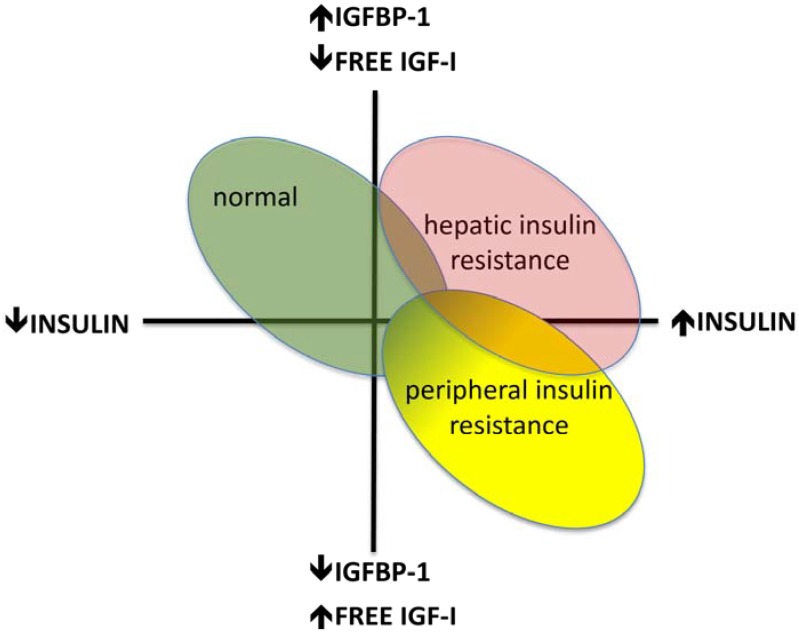
Changing relationships between fasting IGFBP-1 and insulin concentrations, and bioavailable (free) IGF-I in tissues in peripheral and hepatic insulin resistance.

IGFBP-1 concentrations are low in hepatic steatosis both in adults [63] and in children [49]. In the progression to type 2 diabetes, IGFBP-1 is less suppressed and is inappropriately high in relation to the prevailing insulin concentrations [16,17]. This may be due in part to reduced hepatic insulin sensitivity. Pro-inflammatory cytokines that accompany the chronic inflammatory state may also contribute to this increase in IGFBP-1 as well as to lower total IGF-I concentrations and relatively lower fIGF-I (Figure 2). Changes in hepatic insulin clearance may also affect this relationship [64].

Several recent reviews explore the links between obesity, insulin resistance and the metabolic syndrome, and cancer [65,66,67,68] highlighting the potential roles of hyperinsulinaemia, higher bioactivity of IGF-I, IGF-II and insulin through IR-A and IGF1R. An exploration of these links is beyond the scope of this review.

## 4. Impaired Glucose Tolerance and Type 2 Diabetes

Fasting IGFBP-1 is a good predictive marker of abnormal glucose homeostasis, with low values predicting the development of impaired fasting glucose, impaired glucose tolerance and type 2 diabetes 8–17 years later [16,17,69,70]. In women who go on develop diabetes 8 years later, IGFBP-1 concentrations are low relative to insulin levels, perhaps due to a novel inhibitor of IGFBP-1. In both men and women, at the time of diagnosis of type 2 diabetes, fasting IGFBP-1 may rise in relation to insulin, which may be in part due to hepatic insulin resistance (Figure 2). Impaired IGFBP-1 suppression in response to an oral glucose load is also a predictor, and may indicate hepatic insulin resistance; however it is not a better marker than the fasting concentrations [17,71]. Low IGFBP-2 and higher IGFBP-3 concentrations are also associated with incident diabetes in middle-aged women [70].

Low IGF-I concentrations have been shown to predict later glucose intolerance and type 2 diabetes in some studies [72] and not others [16,17,69]. Free IGF-I concentrations are associated with incident diabetes in women with insulin levels above, and IGFBP-1 levels below, the median [70].

Poorly controlled type 2 diabetes is associated with an increase in IGFBP-3 glycation, which increases affinity for IGF-I, as well as additional sialylation that has the converse effect, to decrease IGF-I affinity [73]. Altered patterns of IGFBP-1 multimerisation have been reported in type 2 diabetes [74]. Levels of soluble IGF2R in the circulation are increased [6,75].

There is evidence to suggest that the IGF system, through effects on cell growth, metabolism and survival, plays a pathophysiological role in the cardiovascular complications of diabetes mellitus [76,77]. It may also play a role in microvascular complications. IGFBP-3 appears to have protective effects on retinal vasculature, and may be a therapeutic candidate in retinopathy [78]. Polymorphisms in IGFBP-1 are associated with diabetic nephropathy [79]. It has been suggested that IGF2BP2 might interact with IGF-II mRNA to protect males with type 1 diabetes from diabetic nephropathy [80].

## 5. Conclusions

The IGF system (GH, IGFs, IGFBPs and their receptors), acting in concert with insulin and other hormone axes, is important in normal metabolism. Obesity is associated with reduced GH secretion that contributes to a shift toward visceral adiposity, while total IGF-I levels are relatively unchanged due to increased hepatic GH sensitivity. Insulin plays a key role in these changes and also inhibits IGFBP-1: low levels predict the development of disturbances in glucose homeostasis including type 2 diabetes several years later. Visceral adiposity, hepatic insulin resistance and chronic inflammation in obesity and type 2 diabetes have an impact on the IGF system that reflects the heterogeneity of these disorders and includes an altered relationship between IGFBP-1 and insulin, leading to IGFBP-1 concentrations that are inappropriately normal or elevated. The IGF system is implicated in the vascular and other complications of these disorders and is therefore a potential therapeutic target.
